# Condition-dependent expression of pre- and postcopulatory sexual traits in guppies

**DOI:** 10.1002/ece3.632

**Published:** 2013-06-05

**Authors:** Md Moshiur Rahman, Jennifer L Kelley, Jonathan P Evans

**Affiliations:** Centre for Evolutionary Biology, School of Animal Biology, University of Western AustraliaCrawley, 6009, Western Australia, Australia

**Keywords:** Carotenoids, dietary manipulation, genetic variation, lek paradox, trade-offs

## Abstract

Female choice can impose persistent directional selection on male sexually selected traits, yet such traits often exhibit high levels of phenotypic variation. One explanation for this paradox is that if sexually selected traits are costly, only the fittest males are able to acquire and allocate the resources required for their expression. Furthermore, because male condition is dependent on resource allocation, condition dependence in sexual traits is expected to underlie trade-offs between reproduction and other life-history functions. In this study we test these ideas by experimentally manipulating diet quality (carotenoid levels) and quantity in the guppy (*Poecilia reticulata*)*,* a livebearing freshwater fish that is an important model for understanding relationships between pre- and post-copulatory sexually selected traits. Specifically, we test for condition dependence in the expression of pre- and postcopulatory sexual traits (behavior, ornamentation, sperm traits) and determine whether diet manipulation mediates relationships among these traits. Consistent with prior work we found a significant effect of diet quantity on the expression of both pre- and postcopulatory male traits; diet-restricted males performed fewer sexual behaviors and exhibited significant reductions in color ornamentation, sperm quality, sperm number, and sperm length than those fed ad libitum. However, contrary to our expectations, we found no significant effect of carotenoid manipulation on the expression of any of these traits, and no evidence for a trade-off in resource allocation between pre- and postcopulatory episodes of sexual selection. Our results further underscore the sensitivity of behavioral, ornamental, and ejaculate traits to dietary stress, and highlight the important role of condition dependence in maintaining the high variability in male sexual traits.

## Introduction

Conspicuous sexual traits that function either in the context of intrasexual interactions or through intersexual mate choice (Darwin 1871) may act as “honest” indicators of male quality. Examples of such traits include weaponry (e.g., horns) used during male–male contests and elaborate ornaments (e.g., colorful plumage, crest, or comb) or courtship songs used to attract females (Andersson 1994). The genic capture hypothesis (see Rowe and Houle 1996) predicts that despite persistent directional sexual selection on such traits, their expression can be highly variable because only individuals of superior quality or condition are able to bear the costs of expressing them (Zahavi 1975; Pomiankowski 1987; Grafen 1990; Iwasa and Pomiankowski 1991, 1999; Cotton et al. 2004a) and because condition itself is underpinned by high genetic variance (reviewed by Tomkins et al. 2004). An individual's condition largely depends on the amount of resources available for acquisition and allocation to fitness-enhancing traits (Lorch et al. 2003; Tomkins et al. 2004); thus condition dependence in secondary sexual traits is thought to underlie trade-offs between costly sexual displays and other life-history functions (i.e., increasing allocation to one function results in decreasing resource availability for alternative functions) (Lozano 1994, 2001; Gustafsson et al. 1995; Griffith 2000; Andersson et al. 2002; Kilpimaa et al. 2004; Peters et al. 2004; McGraw 2006; Simmons and Emlen 2006; Perry and Rowe 2010).

The manipulation of resource availability, particularly through dietary manipulation, offers a useful way of testing for condition dependence in sexually selected traits. Accordingly, many studies have shown that males fed diets of high nutritional quality and/or quantity exhibit enhanced ornamental (Hooper et al. 1999; Hill 2000; Cotton et al. 2004a; Tibbetts 2010; Devigili et al. 2012), behavioral (Gottlander 1987; Mappes et al. 1996; Jennions and Backwell 1998; Kotiaho 2000, 2001; Engqvist and Sauer 2003; Holzer et al. 2003; Kim and Choe 2003; Devigili et al. 2012), and morphological traits (Hunt and Simmons 1997; Meidel and Scheibling 1999; Bjorksten et al. 2000; Laparie et al. 2010). Dietary manipulation may also influence the expression of traits subject to postcopulatory sexual selection, which comprises sperm competition, where ejaculates from two or more males compete for the fertilization of ova (Parker 1970), and cryptic female choice, where females influence the outcome of sperm competition through physiological, morphological, or behavioral adaptations (Thornhill 1983; Eberhard 1996). Because females typically mate with two or more males within a single reproductive episode (Birkhead and Møller 1998), postcopulatory sexual selection is a potent evolutionary force in most mating systems (Birkhead and Pizzari 2002). Studies that have tested for condition dependence in traits subject to postcopulatory sexual selection have revealed evidence that dietary manipulation influences ejaculate size (Kemp et al. 1991; Delisle and Bouchard 1995; Jia et al. 2000; Ferkau and Fischer 2006; Lewis and Wedell 2007; Franssen et al. 2008; Perry and Rowe 2010; Rogers-Bennett et al. 2010), sperm quality (Gage and Cook 1994; Simmons 2012), and testes size (Ward and Simmons 1991). Surprisingly, however, despite the importance of both pre- and postcopulatory episodes of sexual selection in most taxa, only a handful of studies have tested for condition dependence in both pre- and postcopulatory traits simultaneously (Pike et al. 2010; Lewis et al. 2011, 2012; Devigili et al. 2012; Tigreros 2013).

Carotenoids constitute an important source of anti-oxidants that are ingested through the diet and may simultaneously contribute toward the expression of male sexual ornaments and ejaculate traits (Grether et al. 1999; Blount et al. 2001, 2003; Velando et al. 2008; Helfenstein et al. 2010). Indeed, carotenoids are known to influence the expression of numerous traits subject to precopulatory sexual selection in many birds and fishes (Kodric-Brown 1989; Hill 1990; Grether 2000; McGraw and Ardia 2003; Karino and Shinjo 2004; Lindstrom et al. 2009), and therefore condition dependence in these signals is both expected (Zahavi 1975; Andersson 1986; Pomiankowski 1987) and supported by empirical evidence in these groups (Nicoletto 1993; Alonso-Alvarez et al. 2004; Pike et al. 2007; Hill et al. 2009; Lindstrom et al. 2009). There is also evidence that carotenoids may influence the expression of traits subject to postcopulatory sexual selection (Helfenstein et al. 2010; Pike et al. 2010), which in turn may explain why carotenoid-based sexual ornaments and components of sperm quality (Locatello et al. 2006; Helfenstein et al. 2010) or sperm competitiveness (Evans et al. 2003; Helfenstein et al. 2008) are correlated in some species. For example, in the three-spined stickleback *Gasterosteus aculeatus*, the expression of carotenoid-based nuptial coloration is positively correlated with male fertility, and males fed experimentally elevated levels of carotenoids exhibit significantly higher fertilization rates than their counterparts fed a low carotenoid diet (Pike et al. 2010). Similarly, in birds, Helfenstein et al. (2010) found that the ejaculates of male great tits (*Parus major*) were susceptible to oxidative stress, and that when relatively less ornamented males were fed carotenoid-supplemented diets their sperm quality improved, suggesting that dull (unattractive) males were deficient in carotenoid antioxidants.

**Figure 1:**
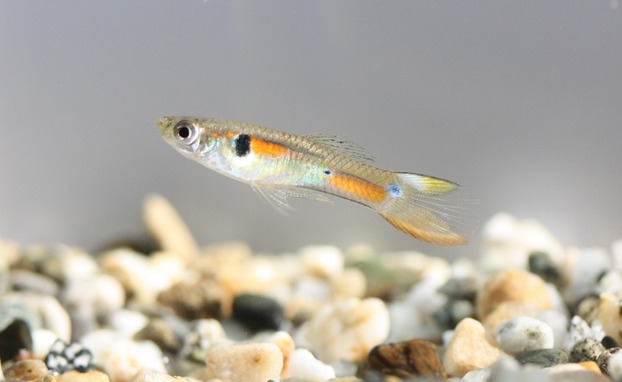
A male guppy (*Poecilia reticulata*). Photograph courtesy of Clelia Gasparini.

The guppy *Poecilia reticulata* (Fig. 1) is an ideal species for evaluating how carotenoids simultaneously contribute toward the expression of pre- and postcopulatory sexual traits. In this polyandrous livebearing fish, males and females exhibit marked sexual dimorphism, with males exhibiting complex color patterns composed of orange (carotenoid and pteridine), iridescent (structural), and black (melanin based) spots and females exhibiting cryptic coloration (Houde 1997). For their part, female guppies exhibit consistent sexual preferences toward males exhibiting relatively large and bright orange spots, which males advertize to females during ritualized courtship displays (Houde 1997). Outside periods of female sexual receptively, males can employ coercive copulation attempts (termed “gonopodial thrusts”), where they attempt to forcibly inseminate females without prior courtship. Several studies on guppies have revealed positive correlations between the extent of orange pigmentation, which exhibits condition dependence in this species (Grether 2000; van Oosterhout et al. 2003; Karino and Shinjo 2004; Kolluru and Grether 2005; Kolluru et al. 2009), and components of sperm quality (Locatello et al. 2006; Pitcher et al. 2007), while two studies have reported a positive association between the extent of a focal male's orange pigmentation and his success in sperm competition (Evans et al. 2003; Pitcher et al. 2003). These latter studies therefore suggest possible functional codependence of pre- and postcopulatory sexual traits on resource availability, which is predicted by theory (Blount 2004; Velando et al. 2008) but so far lacking in direct experimental support.

In this study, we determine whether the experimental manipulation of dietary carotenoids influences the expression of precopulatory (sexual behavior and color ornamentation) and postcopulatory (the velocity, viability, number, and size of sperm) traits in guppies. Our manipulation of carotenoid levels spanned the period from the onset of sexual maturity (around 13 weeks) until males were aged 7 months, and involved the complete exclusion of dietary carotenoids in the restricted group, thus maximizing any impact of carotenoid deficiency on the observed traits. Because the expression of male sexual traits is sensitive to diet quantity (Devigili et al. 2012), we also explored the effects of carotenoid manipulation under experimentally low and high food levels, thus potentially exposing any interactive effects of diet quantity and quality on the expression of pre- and postcopulatory sexual traits. Based on prior work (Devigili et al. 2012) we expected to see a reduction in trait values under food limitation, and interacting effects of carotenoid supplementation and food levels under the assumption that the effects of carotenoid limitation will be more prevalent under dietary stress (Hill et al. 2009). Finally, we test for condition dependence in both sexual and nonsexual (morphological) traits, with the a priori expectation that traits under sexual selection should exhibit greater condition dependence than nonsexually selected traits (Cotton et al. 2004b).

## Methods

### Origin and maintenance of study fish

The guppies used in this experiment were descendents of wild-caught fish from Alligator Creek in Queensland, Australia and were kept in mixed-sex aquaria at the University of Western Australia (115 L tanks) until required for this experiment. These stock tanks were lit by overhead fluorescent lamps (Philips TLD 36W, Royal Philips Electronics, Amsterdam, the Netherlands) on a 12:12 light: dark cycle and maintained at 27°C. The stock population was fed 6 days per week on a diet of *Artemia* nauplii which was supplemented with commercial dry food 3 days per week (Wardley Total Tropical Gourmet Flake Blend™, The Hartz Mountain Corporation, Secaucus, NJ).

### Dietary treatments

The males used in this experiment were aged 3 months at the start of the trials. These males (*n* = 120 in total) were assigned at random to one of two experimental diet treatments (hereafter termed “high quality” and “low quality”), and then further divided into two food level treatments (“high quantity” and “low quantity”). Our experimental design therefore tested the effect of two factors (quality and quantity) and their interaction on male traits, with *n* = 30 males assigned to each of the four diets. The two experimental diets (i.e., high and low quality) were prepared as “micro-pellets” (pellet size: 300–500 μm) by NutraKol Pty Ltd, Western Australia (see Table 1 for diet formulations); the high-quality (carotenoid-enriched) diet was identical to the low-quality diet except for the presence of four carotenoid pigments (i.e., they were nutritionally identical in other regards; see Table 1). Both diets were compositionally similar to those used in previous guppy studies (Grether 2000, 2005; Kolluru et al. 2006) and based on naturally occurring levels of these pigments in the diet of wild fish (Grether et al. 1999, 2001).

**Table 1:** Composition of high- and low-quality experimental diets (dry weight basis)

Diets	Ingredients					Carotenoid supplements (μg/g)			
	
	Fish and mussel meal^1^	Fish oil	Vitamins^2^	Gelatin	Others^3^	Lutein	Zeaxanthin	Astaxanthin	ß-carotene
Low	74.8	5.9	3.5	5.2	10.6	0	0	0	0
High	74.8	5.9	3.5	5.2	10.6	1000	100	100	1500

1 Fish meal: 55% and mussel meal: 19.8%.

2 Vitamin C: 1.2%; vitamin E: 0.7%, and vitamin mix: 1.6%.

3 Lecithin: 1.2%; immune stimulant: 3.5%, and egg white: 5.9%.

Once assigned to their allotted diet treatments, males were reared individually in 2 L tanks (illuminated on a 12:12 h cycle with Philips TLD 36W fluorescent lamps) and fed standard amounts of the micro-pellets once daily (6 days per week) at a rate of ∼4% (1.9 mg; high quantity) or ∼2% (0.9 mg; low quantity) of their body weight until they were sexually mature (7 months old). Rearing males individually and in random positions in a temperature-controlled room (mean 26°C; range 25–27°C) ensured that common environmental effects would not have contributed to any of the observed differences in trait values in this experiment. During the 4-month rearing period 13 males died prior to testing (*n* = 3 in high-quality/high-quantity group; *n* = 3 in low-quality/high-quantity group; *n* = 5 in high-quality/low-quantity group and *n* = 2 in low-quality/high-quantity group).

As male guppies exhibit largely determinate growth (i.e., their size does not change substantially after reaching sexual maturity), food levels were maintained at these levels throughout the experimental period. We standardized the quantity of micro-pellets by using an electric balance to ensure that food quantities did not differ among males within each group throughout the feeding trials. We chose the amount given following preliminary trials, which confirmed that most fish assigned to the low-quantity diet consumed their food within 10 min, while those assigned to the high-quantity group continued feeding well beyond this time. During the 4-month rearing phase, each focal male had visual (but not direct) access to two adult females housed in adjacent tanks to ensure that they remained sexually motivated (e.g., see Bozynski and Liley 2003; Gasparini et al. 2009). Opaque paper screens were placed between adjacent male tanks to prevent visual interactions among the experimental males. In all tanks, the water was partially exchanged (30–40%) and treated with an antialgal treatment (2-chloro-4, 6-bis-(ethylamino)-s-triazine) each week to prevent algal growth, which may otherwise provide a source of carotenoids to the experimental fish (Grether 2000).

### Mating behavior

Mating behavior trials took place between 0800 and 1200 to correspond with the peak of sexual activity in this species (Houde 1997). For these trials, we used ten replicate 8 L observation tanks (28.5 × 14.5 × 19 cm, filled to 14 cm) containing aquarium gravel and artificial pondweed. In each trial, a nonvirgin female from a mixed-sex (stock) aquarium was placed in the tank and allowed to settle overnight. Females were approximately matched for size (by eye) across trials and used only once. The following day, a single male was placed in the aquarium and allowed to settle for at least 5 min or until it showed sexual interest in the female (i.e., following the female or engaging in courtship). For each 15 min trial, we recorded male mating behavior as the number of sigmoid displays (males arch their body in a characteristic s-shaped posture and quiver), gonopodial thrusts (forced mating attempts in which males approach females from behind and attempt copulations without prior courtship or female solicitation), and the time (in seconds) spent by the male courting or chasing the female (a measure of the male's overall sexual interest in the female, hereafter “sexual interest”). After the trial, each male was returned to its individual tank and maintained on the same diet treatment for a further 7 days before being used for the body size, color pattern, and sperm analyses. This period of isolation after the mating behavior trials ensured that males would have fully replenished their sperm supplies prior to sperm counts and sperm analyses (see below) (Bozynski and Liley 2003; Gasparini et al. 2009).

### Male body size and coloration

One week after the behavioral trials, each male was anesthetized and its body surface was gently dried with blotting paper. The male was then photographed under standard lighting (two 13W fluorescent bench lamps) against a measurement scale on a white background using a digital camera (Nikon D70s with Nikon 105 mm macro lens, Nikon Corporation, Tokyo, Japan). Each image included a color standard (mini Munsell Colour Checker™, Munsell Color, Grand Rapids, MI) and a unique code so that subsequent analyses of male traits were performed blind of treatment. The raw uncompressed images (.NEF) were converted to TIFF files and imported into *ImageJ* software (http://rsbweb.nih.gov/ij/) for the measurements of body size and coloration. We measured the total area of the male's colored spots on the left side of the body, including the area of carotenoid and pteridine pigments (orange and yellow spots, hereafter summed as “orange”) and structural colors (blue, green, purple, silver, and white, collectively termed “iridescence”; Brooks and Endler 2001b). The total number of orange and iridescent spots was also recorded as an index of color complexity (Nicoletto 1993; Brooks and Endler 2001a). Standard length (the distance in mm from the male's snout to the tip of his caudal peduncle) and body area (the area [mm^2^] of the body excluding all fins) was estimated to test for a possible trade-off between somatic growth and sperm quality.

The reflectance of each male's colored spots was measured directly from the digital photographs using ColourWorker software (developed by John Anderson and Daniel Osorio, University of Sussex, U.K.; Chrometics Ltd.: http://www.chrometrics.com/download.html). This program utilizes reference spectra that are specific to the reflectance properties of the subject material being photographed and compares this to the spectral information obtained from a color standard (with known reflectance properties), which is included in each image. The inclusion of the color standard in every digital photograph allows the program to compensate for any variation in reflectance due to ambient lighting or variation in the light encoding capability of the camera. As ColourWorker does not include reference spectra that are suitable for evaluating the reflectance of fish skin pigments, we imported spectra obtained from a previous experiment investigating the influence of diet quantity on male guppy coloration. In this previous experiment (Devigili et al. 2012), guppies originated from the same Queensland population as those used in the current experiment and skin reflectance was measured using a USB4000 spectrometer (Ocean Optics, Inc., Dunedin, FL) and miniature Deuterium Tungsten light source (Analytical Instrument Systems, Inc., NJ) (see Devigili et al. 2012 for full details of spectrometry). Spectral data were collated using SpectraSuite software (Ocean Optics), linearly interpolated at 5 nm intervals (range: 400–700 nm) and then imported as reference files into the ColourWorker program (*n* = 60 orange spectra and 30 iridescent spectra).

We used ColourWorker to obtain three measures of reflectance for each color spot by placing the program's point sampler in a different location (but toward the center of each patch) within each color spot. Each spot was measured specific to its corresponding reference spectra; thus orange spots were measured by selecting our orange guppy spectra as reference material and iridescent spots were measured using our iridescent guppy reference spectra. We obtained a measure of reflectance (between 0 and 1) for each 5 nm wavelength interval between the range 400–700 nm. For each male we averaged the three reflectance measurements for each spot (for orange and iridescent patches separately). Reflectance data from ColourWorker can only be obtained between 400 and 700 nm; thus we could not obtain measures of ultraviolet (300–400 nm) reflectance using this method.

Finally, principal component analysis (PCA) was used to reduce the number of color pattern reflectance measures (*n* = 61 measures of reflectance per patch) for our subsequent analyses (see below). We performed PCA for the orange and iridescent spectra separately (using all males), entering reflectance at each wavelength (e.g., 400 nm, 405 nm, 410 nm…) as a separate variable. We only considered principal components with eigenvalues greater than one and obtained four PCs for orange spots (OR-PC_1–4_) and three PCs for iridescent spots (IR-PC_1–3_) (Table 2). We determined how the PCs were loaded against the original (wavelength) variables by plotting wavelength against the factor loadings in each case (see Figs. S1, S2). As for other studies that have used PCA for the analysis of spectral data, we confirmed that PC1 (which accounted for >65% of the total variability in the PCA) represents mean spectral reflectance (typically correlated with brightness), while subsequent PCs describe the shape of the reflectance spectrum (which is correlated with hue and chroma) (Cuthill et al. 1999).

**Table 2:** Eigenvalues and proportion of variation explained for the principal components (PCs) of orange spots (OR-PC_1–4_), iridescent spots (IR-PC_1–3_), and original sperm velocity traits (CASA-PC_1–2_)

PCs	Eigenvalues	% of variation explained
Orange spot PCs		
OR-PC1	39.39	64.6
OR-PC2	9.56	15.7
OR-PC3	8.63	14.2
OR-PC4	3.2	5.3
Iridescent spot PCs		
IR-PC1	41.43	67.9
IR-PC2	11.46	18.8
IR-PC3	8.04	13.1
Casa PCs		
CASA-PC1	4.44	63.4
CASA-PC2	1.57	22.5

### Analysis of body shape

**Figure 2:**
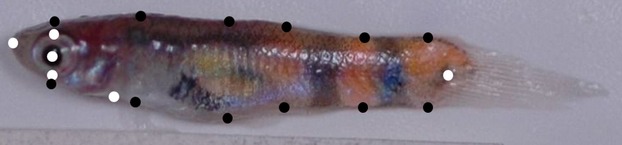
Identification of landmarks used in the geometric morphometric analysis. We used six fixed (white dots) and 12 sliding (black dots) semilandmarks that were positioned on each image using tpsDig2 software.

We used geometric morphometrics to estimate variation in male body shape largely following the methods described by Hendry et al. (2006). Briefly, we used tpsDig2 software (Rohlf 2005a) to superimpose 18 landmarks on each image (see Fig. 2). Landmarks were subsequently designated as fixed (placed at homologous points on each image) or semisliders (placed on curved surfaces between fixed landmarks) using tpsUtil software (Rohlf 2005b). Landmark data were subsequently analyzed using tpsRelw v1.42 software (Rohlf 2005b) to generate relative warp (RW) scores, which describe shape variation as deviations from a consensus shape. These scores were subject to RW analysis, which corresponds to a principal components analysis and serves to reduce multivariate shape data to RWs that describe most of the variation in shape. We retained three RWs, which collectively explained ∼64% of the overall variance in male body shape (hereafter referred to as RW_1–3_). The shape variation captured by these RW scores is illustrated by the thin-plate splines in Figure S3. Briefly, RW_1_ describes variation in the shape of the abdomen (flank), RW_2_ describes variation in the shape of the gill region and operculum plate, and RW_3_ describes the elongation of males’ caudal peduncle. These three indices of body shape were included in our analysis to provide measures for nonsexual “traits” with the expectation that these traits would exhibit reduced condition dependence in comparison to traits subject to sexual selection (Cotton et al. 2004b).

### Sperm assays

Immediately after the digital photography, the anesthetized males were carefully dried and placed on a glass slide under a dissecting microscope with their gonopodium (intromittent organ) swung forward. A micropipette was used to add 40 μL of an extender medium (207 mmol/L NaCl, 5.4 mmol/L KCl, 1.3 mmol/L CaCl_2_, 0.49 mmol/L MgCl_2_, 0.41 mmol/L MgSO_4_, 10 mmol/L Tris, pH 7.5) at the base of the gonopodium. Light pressure was then applied to each male's abdomen to expel all strippable sperm into the extender medium (Matthews et al. 1997). The use of the extender medium ensured that sperm bundles remained intact and quiescent until they were used for the sperm performance assays (Gardiner 1978). From this total sperm pool, we extracted 10 spermatozeugmata (unencapsulated sperm bundles) for the sperm viability assays (see below) before activating the remaining sperm with 40 μL of 150 mmol/L KCl solution with 2 mg/L bovine serum albumin (BSA) (Billard and Cosson 1990). The use of BSA in this solution helped to prevent sperm from sticking to the slide (Pitcher et al. 2007). From these activated sperm samples, two aliquots (each containing three sperm bundles) were immediately used for the computer-assisted sperm analysis (CASA) of motility (see below). Sperm bundles not used for CASA and sperm viability assays were collected in a known volume of saline solution and 1% formalin (to prevent sperm degradation) and stored at 4°C until counted.

#### Computer-assisted sperm analysis

Sperm velocity was estimated immediately after each sample was activated with the KCl/BSA solution using CASA (following Evans 2009). For these assays, the two freshly activated samples from each male were placed in separate wells of a 12-cell multitest slide (MP Biomedicals, Aurora, OH) previously coated with 1% polyvinyl alcohol to further prevent sperm from sticking to the glass slide (Wilson-Leedy and Ingermann 2007). The sample was then analyzed using the CEROS Sperm Tracker (Hamilton Thorne Research, Beverly, MA). The measures included: (1) average path velocity (VAP), which estimates the average velocity of sperm cells over a smoothed cell path; (2) straight line velocity (VSL), the average velocity on a straight line between the start and the end point of the track; (3) curvilinear velocity (VCL), the actual velocity along the trajectory; (4) linearity (LIN), the ratio of net distance moved to total path distance (VSL/VCL); (5) straightness (STR), the ratio of net distance moved to smoothed path distance (VSL/VAP); and (6) the amplitude of lateral head displacement (ALH), the magnitude of lateral displacement of a sperm head about its spatial average trajectory. The threshold values for defining static cells were predetermined at 24.9 μm/sec for VAP and VCL, and 15 μm/sec for VSL (Evans 2009). Sperm velocity measures were based on an average of 35.1 ± 2.09 SE sperm tracks per sample (mean value is taken for *n* = 104 males; *n* = 3 males did not produce sperm).

We used principal components analysis to reduce the number of sperm trait variables (*n* = 7), many of which were highly correlated, to a single composite measure of “speed”, which is known to predict competitive fertilization success in guppies (Boschetto et al. 2011). The first principal component (PC1) from this analysis was strongly positively loaded by VAP, VSL, and VCL, the frequency of tail beating (BCF) and measures of STR and LIN (see Table 3). Thus, collectively PC1 describes the speed and STR of sperm between the start and end point of their tracks.

**Table 3:** Loading matrix with correlations between principal components (PC1–PC2) and original sperm velocity traits

Traits	PC1	PC2
VAP	0.94668	−0.05298
VSL	0.93471	−0.23062
VCL	0.70657	0.63464
ALH	−0.09061	0.96435
BCF	0.66168	0.22074
STR	0.94264	0.09374
LIN	0.91485	−0.36042

The eigenvalues of PC1 and PC2 were 4.44, 1.57 and explained 63.4% and 22.5% of the variation in the data, respectively.

#### Sperm viability

A live/dead sperm viability assay (Invitrogen, Molecular Probes, Life Technologies Corporation, Carlsbad, CA) was used to estimate the proportion of live sperm in the reserved subsample of each male's stripped ejaculate. The assay stains live sperm green with the membrane-permanent nucleic acid stain SYBR-14, and dead sperm red with propidium iodide, which penetrates damaged sperm cell membranes. Sperm bundles were agitated using a pipette and vortex, suspended in 10-μL extender medium and mixed with an equal volume of 1:50 diluted 1 mmol/L SYBR-14. Samples were left in the dark for 10 min before 2 μL of 2.4 mmol/L propidium iodide was added. Samples were incubated in the dark for a further 10 min before being observed under a fluorescence microscope. The proportion of live sperm in each sample was then estimated from 200 sperm cells per sample.

#### Sperm number and length

Sperm counts were performed using an improved Neubauer hemocytometer under 40× magnification (Leica DM1000 microscope, Leica, Solms, Germany) after vortexing each sample for 10 sec. The average of five counts per male was used to estimate the total number of sperm in each stripped ejaculate (Matthews et al. 1997). Sperm counts were corrected to allow for sperm that had been removed from each sample for the CASA and viability assays (Evans 2009).

Photographs of each male's sperm were obtained under 1000× magnification (Leica DM1000 microscope) using a digital camera (Leica DFC320). Where possible, 20 undamaged spermatozoa were analyzed per male (mean number of sperm cells analyzed per male = 19.3 ± 0.25 SE; range = 10–20). *ImageJ* software was used to measure the total length of each sperm cell (Gasparini et al. 2010).

### Statistical analysis

A total of 107 males survived until the end of the experiment. All data exhibited normal distributions with the exception of the sexual behaviors (sexual interest, sigmoid displays, gonopodial thrusts), sperm counts, and sperm viability. Appropriate transformations (log_10_ for all traits except sperm viability, where arcsine square-root transformation was used) yielded normal distributions for all of these traits. We performed a multivariate general linear model (MGLM) to test for an overall effect of the two diet treatments (quality and quantity) and their interaction on precopulatory (Or-PC_1–4_, IR-PC_1–3_, orange area, iridescent area, orange spot number, iridescent spot number, sexual interest, sigmoid displays, gonopodial thrusts), postcopulatory (sperm velocity PC1, sperm viability, sperm counts, total sperm length), and nonsexual traits (body shape RW_1–3_). In this analysis we included male body length (standard length) as a covariate, while the two treatments and their interaction were fitted as fixed effects. As the MGLM revealed an overall significant effect (see below), subsequent univariate general linear models (GLMs) were used to test for the effect of the significant factors on individual traits. For these univariate tests we corrected for multiple comparisons using the False Discovery Rates (FDRs) method proposed by Benjamini and Hochberg (1995). To quantify the magnitude of treatment effects, we calculate Cohen's effect size (Cohen 1988) for each trait. Finally, we calculated Pearson's *r* to test for correlations between pre- and postcopulatory traits. For these tests, we restricted our analysis to traits that were significantly affected by the treatments. We used partial correlations for those traits that were significantly influenced by male standard length. All analyses were performed using JMP® version 9.0.0 (JMP, Cary, NC).

## Results

The overall model for sexual traits (MGLM) revealed a significant effect of diet quantity on the response variables but no effect of diet quality or the quantity-by-quality interaction (Table 4A). Overall, males fed the high-quantity diet were larger than those assigned to the low-quantity treatment (SL mean ± SE; high: 15.84 ± 0.23 mm, low: 14.45 ± 0.09 mm; *F*_1, 104_ = 30.4, *P* < 0.001). The MGLM therefore included male SL as a covariate when testing the overall effects of diet treatment on the sexual and nonsexual traits. The subsequent univariate GLMs revealed that diet quantity had an effect on male sexual behavior, coloration and sperm performance (see below). As predicted, the diet treatments had no significant influence on the first two principal sources of variance in male body shape (RW_1_ and RW_2_). However, there was an effect of diet quantity on RW_3_ (Table 4B), which although unanticipated may reflect the effect of diet restriction on components of body size (see Discussion).

**Table 4:** Results of diet quantity, quality, and their interaction on male sexual traits

(A)			
Response	df	*F*-ratio	*P*
Overall model	20, 59	25.9	<0.001
Diet quantity	20, 56	15.5	<0.001
Diet quality	20, 56	0.55	0.92
Diet quantity × quality interaction	20, 56	0.54	0.94
SL (covariate)	20, 56	2.02	0.020

(B)							
Trait	Response	Mean ± SE		df	*F*	*P*	Effect size (*r*)

		High quantity	Low quantity				
Body shape	RW_1_			1, 87	1.72	0.19	
	RW_2_	n/a	n/a		0.20	0.66	n/a
	RW_3_				5.52	0.021*	
Behavior	Sexual interest	2.65 ± 0.023	2.45 ± 0.057		6.51	0.012*	0.299
	Sigmoid displays	0.959 ± 0.05	0.632 ± 0.072	1, 103	6.72	0.011*	0.338
	Gonopodial thrusts	0.606 ± 0.04	0.552 ± 0.047		0.48	0.490	0.079
Color patterns	Orange PC1	−2.248 ± 0.56	2.248 ± 1.0		16.1	<0.001***	0.356
	Orange PC2	−0.746 ± 0.32	0.746 ± 0. 49	1, 101	7.34	0.008*	0.24
	Orange PC3	0.159 ± 0.31	−0.159 ± 0.48		0.09	0.77	0.053
	Orange PC4	−0.242 ± 0.21	0.242 ± 0.27		3.21	0.08	0.134
	Orange area	5.28 ± 0.294	3.76 ± 0.286		4.98	0.028*	0.341
	Orange spots	5.788 ± 0.21	3.90 ± 0.202		21.8	<0.001***	0.534
	Iridescent PC1	−2.254 ± 0.78	2.254 ± 0.89		9.10	0.003**	0.348
	Iridescent PC2∧	−0.055 ± 0.44	0.055 ± 0.50		3.88	0.051	0.016
	Iridescent PC3	0.873 ± 0.37	−0.873 ± 0.38		7.13	0.009*	0.306
	Iridescent area∧	14.179 ± 0.45	14.305 ± 0.39		7.67	0.007*	0.02
	Iridescent spots∧	10.635 ± 0.31	9.7308 ± 0.35		0.15	0.703	0.183
Sperm traits	CASA PC1	1.459 ± 0.10	−0.967 ± 0.75	1, 100	261.8	<0.001***	0.869
	Sperm viability	0.841 ± 0.008	0.78 ± 0.009	1, 100	12.89	0.001**	0.412
	Sperm number	6.82 ± 0.025	6.45 ± 0.032	1, 94	51.5	<0.001***	0.673
	Sperm length	53.688 ± 0.14	52.843 ± 0.10	1, 96	15.8	<0.001***	0.425

Overall results of the MGLM are given in (A), with male body length (in mm) entered as a covariate and diet quantity, quality, and their interaction as fixed effects. Separate univarate GLMs were subsequently conducted for each of our traits entering diet quantity as a fixed effect and male standard length as a covariate (B). Mean ± SE is not reported for the RW scores as they are standardized to have a mean of zero.

Body length had a significant effect only for those traits marked with ∧(covariate effects: iridescent PC2: *F*_1, 101_ = 9.69, *P* = 0.002; iridescent area: *F*_1, 101_ = 19.3, *P* < 0.001; iridescent spots: *F*_1, 101_ = 4.00, *P* = 0.048)..

Asterisks denote significance after controlling for FDR.

* *P* < 0.05,

** *P* < 0.01,

*** *P* < 0.001; *n*_tests_ = 21.

### Effect of diet on precopulatory sexually selected traits

**Figure 3:**
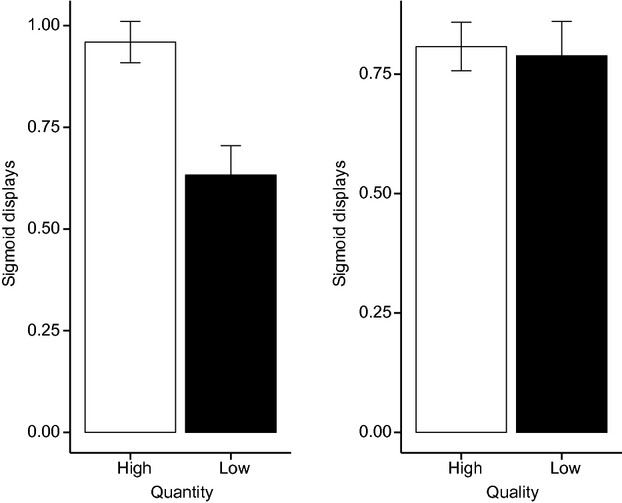
The mean (±SE) sigmoid displays for males fed high- and low-quantity and -quality diets.

**Figure 4:**
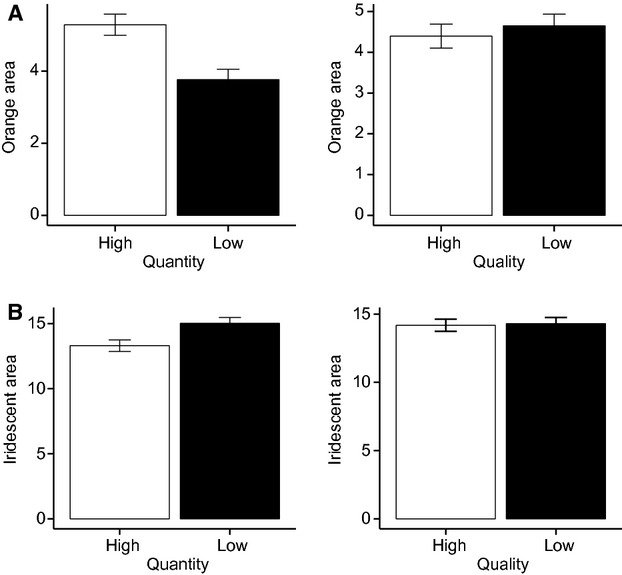
The mean (±SE) orange area (A) and iridescent area (B) for males fed high- and low-quantity and -quality diets.

We found that diet-restricted males performed fewer courtship (sigmoid) displays and exhibited a reduction in sexual interest during the behavioral trials compared with males assigned to the high-quantity group (for the effect of diet treatment on sigmoid displays see Fig. 3). By contrast, we detected no significant difference in the number of gonopodial thrust attempts between the two quantity groups (Table 4B). Male color patterns also differed between the high- and low-quantity groups. Males on the low-quantity diet exhibited a reduction in the area of orange pigmentation and fewer orange spots than those in the high-quantity group (Fig. 4A). However, these food-deprived males had a larger total area of iridescent pigmentation (but not a greater number of iridescent spots) than those in the high-quantity group (Fig. 4B).

**Figure 5:**
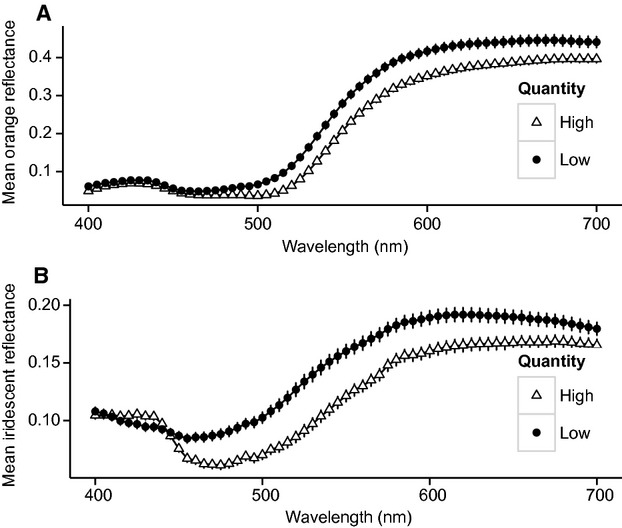
The mean (±SE) spectral reflectance of orange (A) and iridescent (B) spots for male guppies assigned to high- and low- quantity diet groups.

The mean reflectance spectra obtained from the photographs of male orange and iridescent color patches are highly comparable to those reported for other studies of guppies that have used reflectance spectrometry (e.g., Kemp et al. 2009). The PCs describing variation in the spectral characteristics of orange and iridescent spots varied between the diet quantity treatments. PC1 (which was positively correlated with brightness) was significantly higher in males assigned to the low-quantity groups, suggesting that these fish had brighter orange and iridescent spots (Fig. 5A and B). For orange spots, OR-PC_2_ was positive for low-quantity males and negative for high-quantity males. OR-PCs _2__–__4_ represent differences in the relative amounts of medium- to short-wavelength light reflected. This suggests that males in the low-quantity group had a greater proportion of medium-wavelength light reflected (450–575 nm: blue–green) relative to short-wavelength light (violet: 400–450 nm) reflected from their orange spots compared to well-fed males. For iridescent spots, IR-PC_3_ describes the relative amount of violet to blue–green light reflected. Food-deprived males with lower IR-PC_3_ scores therefore also had relatively higher reflectance at medium wavelengths of light (blue–green) compared with short wavelengths of light (violet) for iridescent spots.

### Effect of diet on postcopulatory sexually selected traits

**Figure 6:**
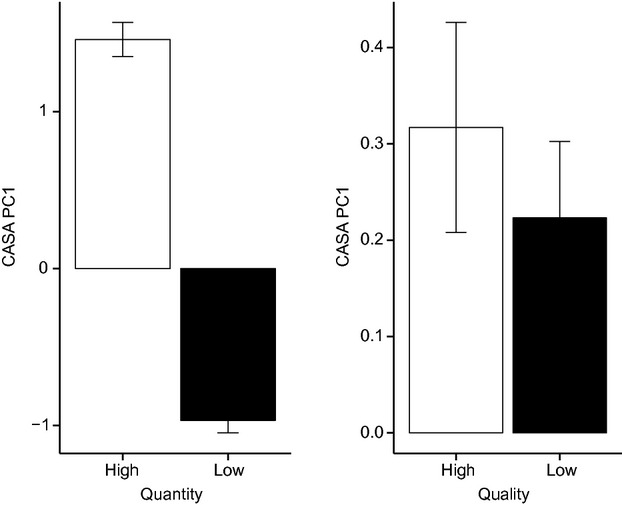
The mean (±SE) PC1 scores from the CASA analysis for males assigned to high- and low-quantity and -quality diets.

All of the sperm traits measured were affected by diet quantity; food-deprived males had fewer, slower swimming, less viable, and shorter sperm than their well-fed counterparts (Table 4B; see also Fig. 6 for PC1 sperm velocity).

### Correlations between pre- and postcopulatory traits

We found no evidence for significant associations (e.g., indicative of trade-offs) between pre- and postcopulatory traits for males maintained on a low-quantity diet (Table 5A). In the high-quantity group, a few negative relationships emerged (Table 5B), but all were rendered highly nonsignificant following correction for FDR.

**Table 5:** Correlations between pre- and postcopulatory traits for males on the low-quantity (A) and high-quantity (B) diets

(A)					
	Precopulatory traits	Postcopulatory traits			
		
	Trait	CASA PC1	Sperm viability	Sperm number	Sperm length
Behavior	Sexual interest	0.14, 51 (0.33)	−0.03, 51 (0.85)	−0.11, 49 (0.46)	0.12, 49 (0.42)
	Sigmoid displays	−0.20, 51 (0.16)	−0.06, 51 (0.66)	−0.03, 49 (0.84)	−0.16, 49 (0.27)
Coloration	Orange PC1	−0.02, 50 (0.91)	−0.14, 50 (0.33)	0.06, 48 (0.67)	−0.02, 48 (0.88)
	Orange PC2	0.02, 50 (0.90)	−0.13, 50 (0.37)	0.20, 48 (0.18)	0.09, 48 (0.53)
	Orange area	−0.10, 50 (0.51)	−0.05, 50 (0.73)	−0.17, 48 (0.25)	−0.18, 48 (0.22)
	Orange spots	−0.09, 50 (0.55)	0.00, 50 (0.98)	−0.20, 48 (0.18)	−0.21, 48 (0.15)
	Iridescent PC1	−0.15, 50 (0.29)	0.15, 50 (0.31)	0.03, 48 (0.86)	−0.19, 48 (0.19)
	Iridescent PC3	−0.20, 50 (0.16)	−0.04, 50 (0.76)	−0.19, 48 (0.20)	−0.19, 48 (0.21)
	Iridescent area^1^	−0.06, 50 (0.66)	−0.17, 50 (0.24)	0.06, 48 (0.70)	−0.09, 48 (0.57)

(B)					
	Precopulatory traits	Postcopulatory traits			
		
	Trait	CASA PC1	Sperm viability	Sperm number	Sperm length
Behavior	Sexual interest	−0.17, 53 (0.21)	−0.12, 53 (0.41)	0.05, 49 (0.72)	−0.03, 51 (0.83)
	Sigmoid displays	−0.19, 53 (0.18)	−0.08, 53 (0.58)	−0.14, 49 (0.35)	0.10, 51 (0.47)
Coloration	Orange PC1	0.14, 51 (0.32)	−0.09, 51 (0.54)	−0.01, 47 (0.93)	−0.18, 49 (0.20)
	Orange PC2	0.24, 51 (0.08)	0.27, 51 (0.05)	−0.05, 47 (0.75)	0.03, 49 (0.86)
	Orange area	−**0.28, 51 (0.049)**	−0.16, 51 (0.25)	−0.08, 47 (0.58)	−0.07, 49 (0.62)
	Orange spots	−0.27, 51 (0.051)	0.01, 51 (0.92)	−0.19, 47 (0.20)	−0.03, 49 (0.85)
	Iridescent PC1	0.05, 51 (0.71)	−0.08, 51 (0.59)	−0.07, 47 (0.64)	−0.20, 49 (0.16)
	Iridescent PC3	−0.05, 51 (0.75)	−0.09, 51 (0.51)	−0.20, 47 (0.18)	−**0.30, 49 (0.040)**
	Iridescent area^1^	**0.29, 51 (0.035)**	−0.01, 51 (0.94)	**0.31, 47 (0.029)**	−0.25, 49 (0.08)

Values shown are correlation coefficients, sample size, and *P*-values (in brackets). Significant values are given in bold but these were all nonsignificant following correction for FDR.

1 Indicates trait analyzed using partial correlations to control for male SL.

## Discussion

Our results reveal that dietary manipulations had a significant effect on the expression of pre- and postcopulatory sexually selected traits, and one component of shape (RW_3_), in male guppies. For precopulatory sexually selected traits, we found that males fed a restricted diet exhibited reductions in courtship, sexual interest, area of orange spots, and orange spot number compared to those assigned to the high-quantity group. The significant effect of diet treatment on one of the nonsexual traits (RW_3_) was unanticipated, given the expectation of relatively lower condition dependence in traits not subject to sexual selection (Cotton et al. 2004b). However, inspection of the thin-plate splines for RW_3_ (Fig. S3) suggests that variation in this component of shape describes the elongation of the male's caudal peduncle, and while this measure was not significantly correlated with SL (*r* = 0.072, *n* = 90, *P* = 0.49), it nevertheless appears to describe variation in the size of this component of the male's body and thus may reflect the overall effect of food limitation on body size.

Our results also revealed strong effects of diet quantity on all ejaculate traits measured in this study. We found that food-deprived males produced fewer and shorter sperm with slower swimming velocities and reduced viability than those assigned to the ad libitum diet treatment. By contrast, we found that diet quality, as manipulated by carotenoid levels, had no effect on the expression of either pre- or postcopulatory sexually selected traits. Furthermore, and contrary to our initial predictions, we found no evidence that males trade resources between pre- and postcopulatory sexual selection, despite evidence from the same population for a (negative) genetic correlation between ejaculate quality and male sexual ornamentation (Evans 2010). Below, we discuss each of these key findings in detail.

### Effect of diet on precopulatory traits

We found a significant effect of diet quantity treatment on components of male courtship but not gonopodial thrusting. Consistent with prior work, we observed a significant reduction in sigmoid displays in the restricted diet group (Devigili et al. 2012), suggesting that this component of male sexual behavior exhibits condition dependence and therefore may provide an honest signal of male quality (van Oosterhout et al. 2003; Kolluru and Grether 2005; Kolluru et al. 2009; Head et al. 2010). Our results also revealed that male sexual interest was influenced by diet (see also Kolluru et al. 2009; Albo et al. 2011; Devigili et al. 2012), suggesting that in guppies, as with some other species, courtship effort is a reliable indicator of male condition (e.g., Shine and Mason 2005; Shamble et al. 2009; Kloskowski et al. 2012). Furthermore, as with Devigili et al.'s (2012) study, our results revealed no significant effect of diet quantity on the frequency of gonopodial thrusts. While this result may indicate that gonopodial thrusts entail relatively lower costs than sigmoid displays (Houde 1997), it is also consistent with the possibility that males redirect limiting resources to alternative reproductive tactics because males in poor condition are unattractive to females irrespective of the level of courtship (see Devigili et al. 2012). Unlike previous work revealing an effect of carotenoid supplementation on the expression of male behavioral traits (van Hout et al. 2011), including guppies (Kodric-Brown 1989; Hill 1991; Grether 2000), we found no such evidence in our study (see also Toomey and McGraw 2012).

Our finding that diet quantity affected the overall area of orange pigmentation and the number of orange spots is consistent with previous work on the same population of guppies (Devigili et al. 2012) and other species (Hill 2000; Tibbetts 2010). Surprisingly, we also found that the total area of iridescent coloration was significantly larger in food-deprived males than their well-fed counterparts. One possible explanation for this latter finding is that a reduction in the area of orange coloration makes the surrounding iridescence patches appear larger and more prominent. Although we found no evidence for an effect of diet quality on male coloration, the observed differences in the spectral properties of orange spots in males fed high- and low-quantity diets is consistent with changes in the carotenoid or drosopterin content of these patches. Specifically, our finding that males fed a high-quantity diet showed a change in wavelength-specific reflectance (chroma) and a reduction in overall spot brightness is in accordance with the absorbance characteristics of carotenoid and drosopterin pigments (Grether 2000) and suggests that these increased in the spots of well-fed males. Although carotenoids are primarily obtained directly through unicellular algae present in the diet (Goodwin 1984), drosopterins can be synthesized from carbohydrates and amino acids (Grether et al. 1999); increased availability of these compounds in males on high-quantity diets may therefore have contributed to the observed changes in the spectral properties of the orange spots.

A further possible explanation for the lack of effect of diet quality in our study is that all males were initially reared to sexual maturity (until 3 months) on a common diet of live brine shrimp (*Artemia*), which are known to contain carotenoids (Gilchrist and Green 1960; Krinsky 1965; Nelis et al. 1988). Thus, carotenoids may not have been sufficiently limited in the low-quality treatment and future studies investigating the effect of dietary carotenoid intake may benefit by introducing dietary manipulations from birth (see Grether 2000). Another explanation is that the skin shrinkage associated with the body area reduction observed in food-limited males may have compensated for any reduced concentration of carotenoids in the pigmented area, causing a reduction of spot size but balancing the reduction in the quantity of carotenoids available for male ornamentation (see also Devigili et al. 2012). Finally, the absorption and bioconversion of some carotenoids are markedly reduced when the intake of fat is low (Jialal et al. 1991; Prince and Frisoli 1993), and therefore a minimum amount of fat is necessary for uptake of carotenoids (Castenmiller and West 1998). Our ongoing work addresses this issue by combining manipulations of carotenoids with dietary fat intake.

### Effect of diet on postcopulatory traits

We found consistent effects of diet quantity on ejaculate quality (e.g., sperm swimming velocity, sperm number, sperm viability, and sperm length), thus confirming condition dependence in these traits. The first principal component for the CASA measures (PC1), which broadly described variation in sperm swimming velocity and STR, was significantly lower in the low-quantity group compared to the high-quantity group (see Fig. 6). Similar to our findings, Selvaraju et al. (2012) found that rams fed a low-energy diet exhibited a significant reduction in sperm motility compared to those fed a high-energy diet, while Alavi et al. (2009) reported that in the cyprinid fish *Barbus barbus*, males fed a diet containing low quantities of polyunsaturated fatty acids (PUFAs) produced ejaculates with lower sperm velocity than those fed a high-quality diet. Furthermore, recent work by Helfenstein et al. (2010) on great tits (*Parus major*) revealed that cartotenoid-deprived pale males produced ejaculates with a lower percentage of motile sperm and spermatozoa with reduced swimming ability compared to males fed a high carotenoid diet. Although we found no effect of carotenoids on sperm velocity, our findings for food quantity, which also extend to measures of sperm viability, provide further support for the conclusion that components of sperm quality are sensitive to dietary stress.

Interestingly, we also detected a significant effect of diet quantity on total sperm length; males assigned to the low-quantity diet had comparatively shorter sperm than their counterparts fed ad libitum. This finding adds to accumulating evidence from other taxa revealing that sperm length may be compromised by male condition. For example, Merrells et al. (2009) found that zinc-deficient rats produced abnormal sperm with relatively short flagella, while Alavi et al. (2009) reported that PUFA deficient male *Barbus barbus* produced relatively short sperm compared to those fed a control diet. Similarly, Immler et al. (2010) noted that significant changes in stress and sex steroid hormone levels influenced sperm morphometry in Gouldian finches. Our results also reveal evidence for condition dependence in sperm numbers, corresponding with several studies on other taxa (Gage and Cook 1994; Simmons and Kotiaho 2002; Perry and Rowe 2010; Selvaraju et al. 2012). Previous work on guppies has revealed positive phenotypic correlations between sperm length and/or sperm numbers and body size (Pilastro and Bisazza 1999; Pitcher and Evans 2001; Skinner and Watt 2007). In our study, males assigned to the high-quantity group were significantly larger than those in the low-quantity group, thus potentially explaining why larger males produced larger ejaculates and longer sperm.

In contrast to our results for diet quantity, we found no significant effect of diet quality on any of the measured postcopulatory traits. As we have noted above in reference to precopulatory traits, one explanation for this finding is that carotenoid-deprived males may have relied on previously ingested pigments obtained prior to reaching sexual maturity (i.e., from *Artemia*). Another explanation may be that carotenoids need to be incorporated with a minimum level of PUFAs or vitamins (A or E) to influence those traits (Castenmiller and West 1998; Almbro et al. 2011).

### Exposing resource allocation trade-offs

Throughout this experiment, resources were limited both quantitatively and qualitatively, yet we found no evidence that dietary manipulation exposes a trade-off between pre- and postcopulatory traits. Although such trade-offs are expected under conditions of resource limitation (van Noordwijk and Dejong 1986), our findings are consistent with several previous studies on guppies, suggesting that males invest equally in pre- and postcopulatory traits so that these mechanisms of sexual selection act in concordance (Evans and Magurran 2001; Evans et al. 2003; Pitcher et al. 2003; Pilastro et al. 2004; Devigili et al. 2012). Nevertheless, trade-offs between pre- and postcopulatory traits might yet be exposed through manipulations of other resources, and one idea we are currently pursuing is to determine whether the interaction between carotenoids and other dietary components affects the relative investment in pre- and postcopulatory sexual selection in this species.

## Supporting Information

**Figure S1.** Factor loadings plotted against wavelength for PC1–PC4 that were used to describe variation in the reflectance spectra of male guppy orange spots. These data illustrate how the loadings of the principal components vary in relation to wavelength. PC1 is loaded positively across the full wavelength range and corresponds to orange spot brightness. PC2 is loaded positively in the wavelength range 475–575 nm and negatively in the range 400–450 nm and represents the relative amount of medium to short wavelength light reflected. PC3 is the amount of short-medium (425–525 nm) to medium-long (525–650 nm) wavelength light reflected while PC4 is weakly positively loaded below 425 nm and between 500–575 nm.

**Figure S2.** Principal component loadings for PC1–PC3 plotted against wavelength for male guppy iridescent spots. As for guppy orange spots, PC1 is positively loaded across all wavelengths and corresponds with spot brightness. PC2 represents the relative amount of short-medium (400–525 nm) to long (>550 nm) wavelength light reflected while PC3 corresponds to the amount of short (<450 nm) and long (>575 nm) wavelength light reflected relative to medium (450–575 nm) wavelength light reflected.

**Figure S3.** The thin-plate splines illustrating shape variation between high (red dots) and low (blue dots) quantity male guppy that were captured by the relative warp (RWs) scores. RW_1_ describes variation in the shape of the abdomen (flank), RW_2_ describes variation in the shape of the gill region and operculum plate, and RW_3_ describes the elongation of males’ caudal peduncle.
