# Supplementary material for: Condition-dependent expression of pre- and postcopulatory sexual traits in guppies
Source: Ecol Evol. 2013 Jun 5;3(7):2197–213. doi: 10.1002/ece3.632 (PMC3728957; doi:10.1002/ece3.632)
Supplement: Supplementary file 1 [file ece30003-2197-SD1.docx]

**Electronic Online Supplementary Material**

**Condition-dependent expression of pre- and postcopulatory sexual traits in guppies**

MD. MOSHIUR RAHMAN, JENNIFER L. KELLEY & JONATHAN P. EVANS

**
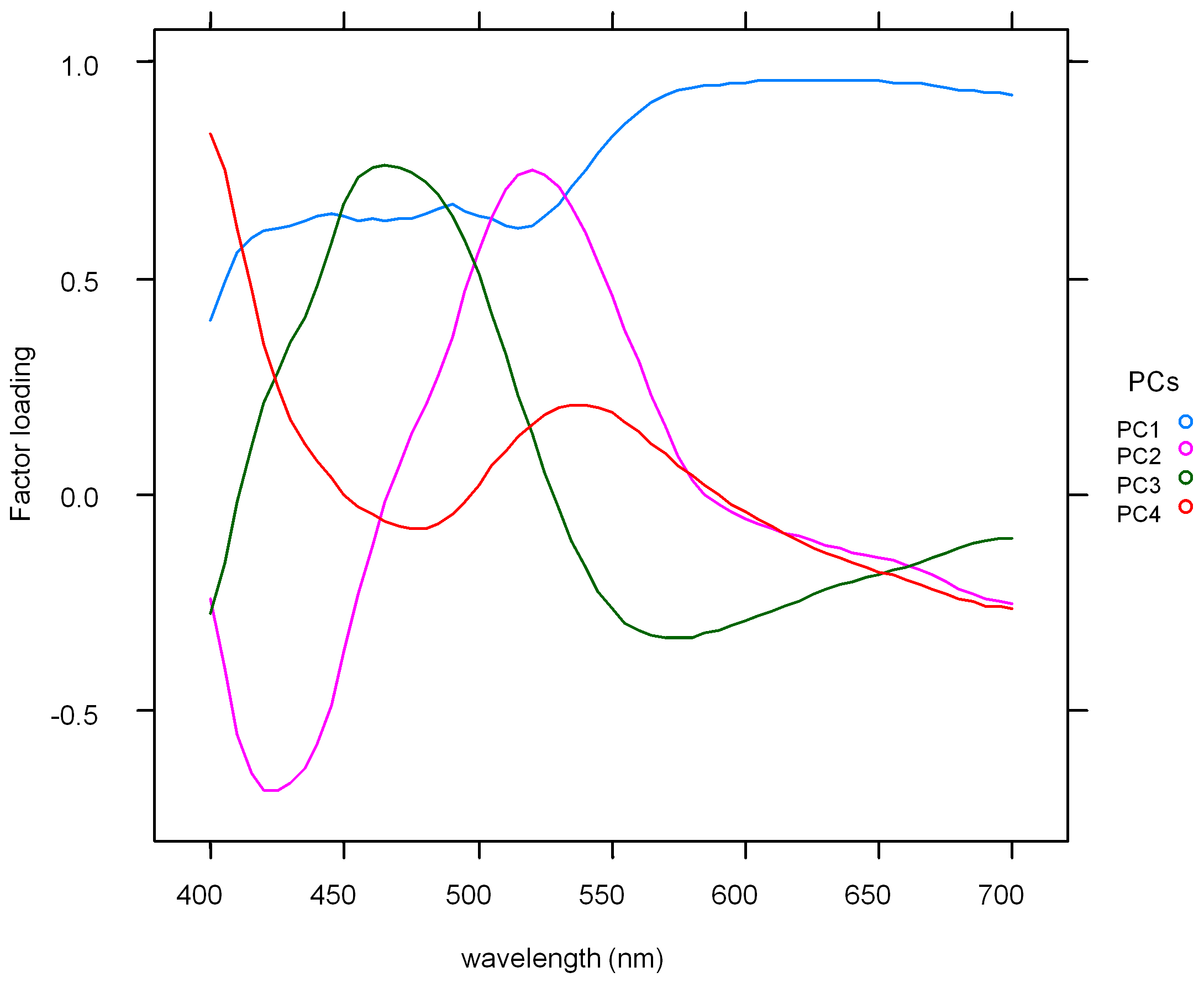
**

**Fig. S1** Factor loadings plotted against wavelength for PC1-PC4 that were used to describe variation in the reflectance spectra of male guppy orange spots. These data illustrate how the loadings of the principal components vary in relation to wavelength. PC1 is loaded positively across the full wavelength range and corresponds to orange spot brightness. PC2 is loaded positively in the wavelength range 475-575nm and negatively in the range 400-450nm and represents the relative amount of medium to short wavelength light reflected. PC3 is the amount of short-medium (425-525nm) to medium-long (525-650nm) wavelength light reflected while PC4 is weakly positively loaded below 425nm and between 500-575nm.


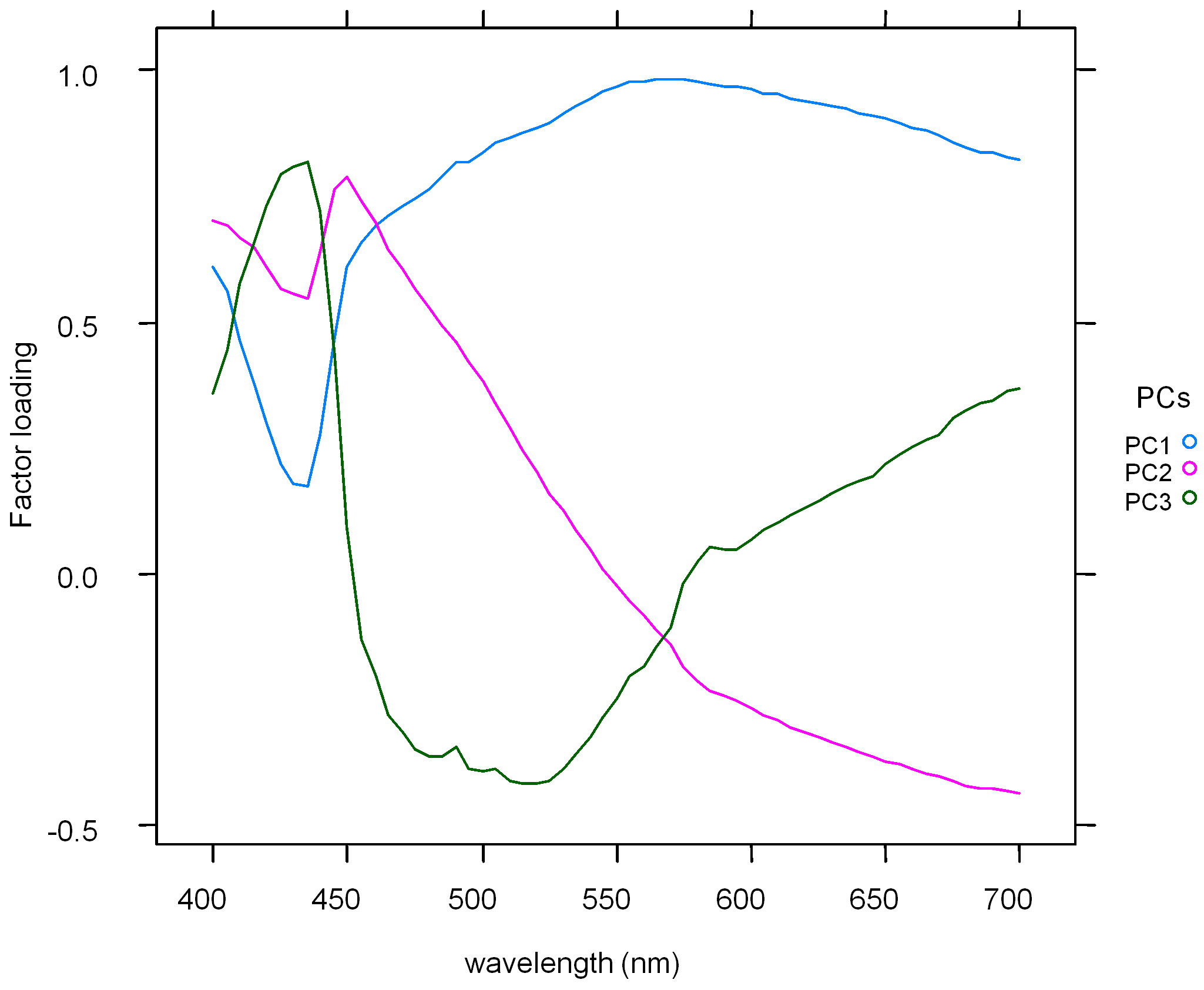


**Fig. S2** Principal component loadings for PC1-PC3 plotted against wavelength for male guppy iridescent spots. As for guppy orange spots, PC1 is positively loaded across all wavelengths and corresponds with spot brightness. PC2 represents the relative amount of short-medium (400-525nm) to long (>550nm) wavelength light reflected while PC3 corresponds to the amount of short (<450nm) and long (>575nm) wavelength light reflected relative to medium (450-575nm) wavelength light reflected.

**
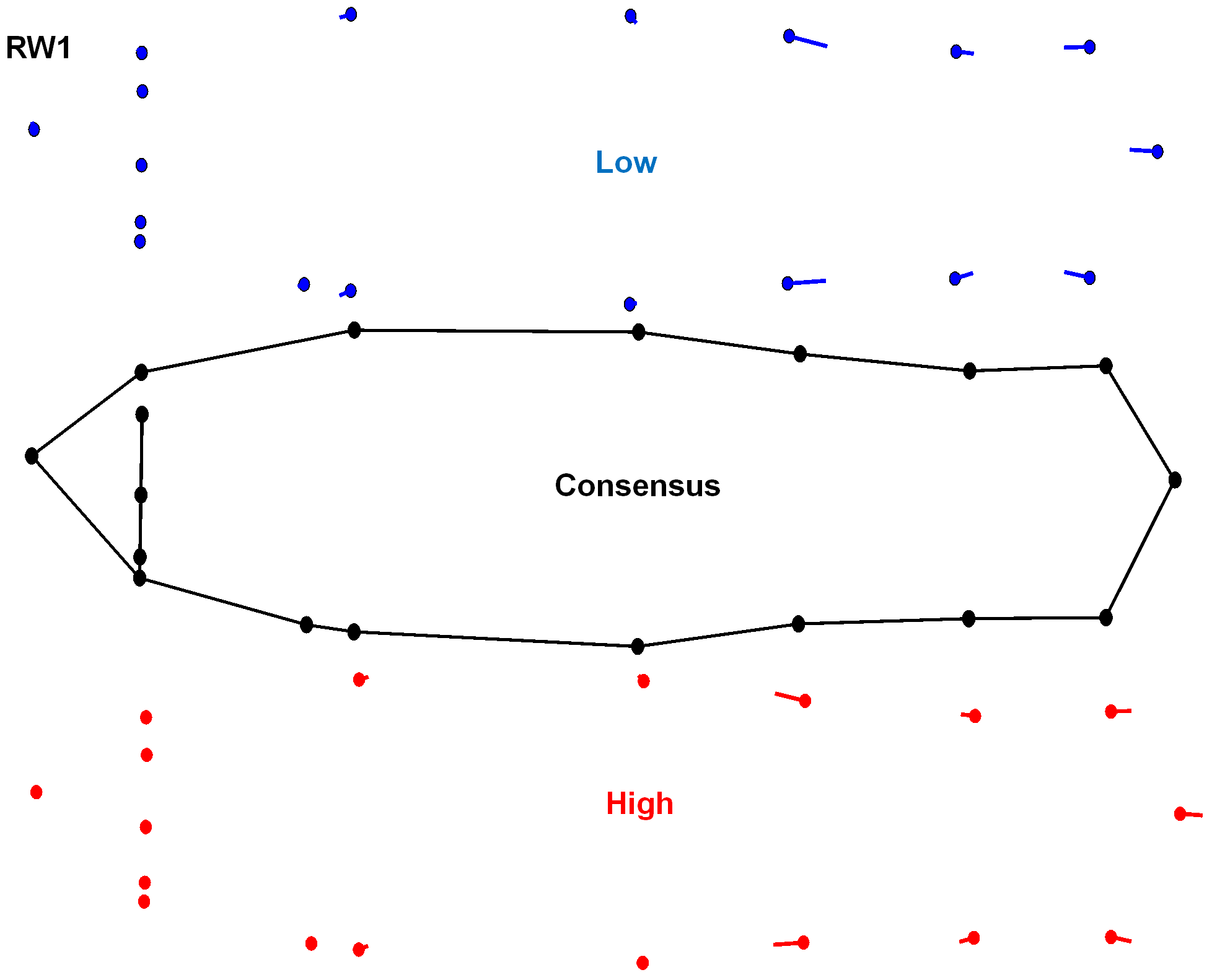
**

**
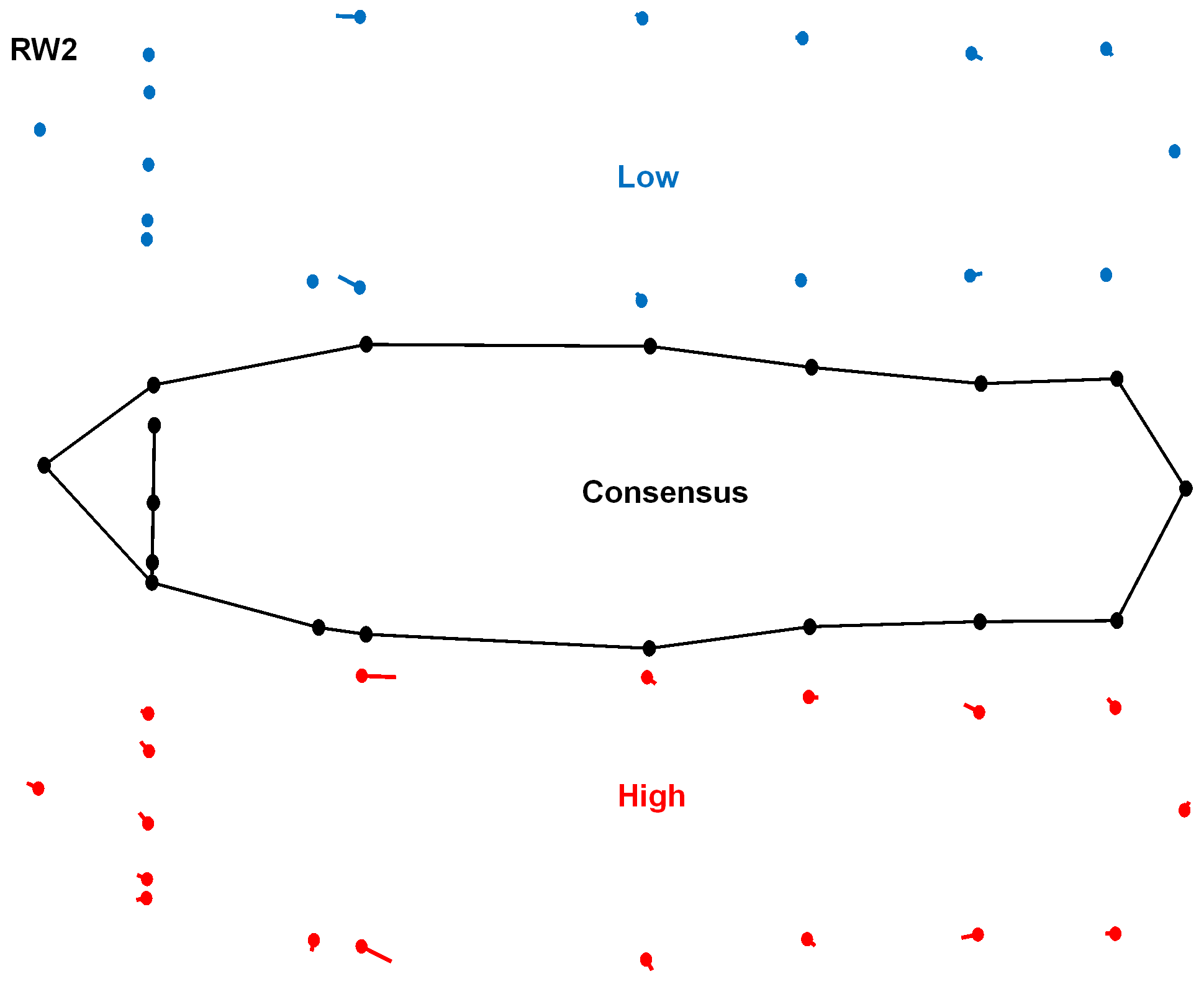
**

**
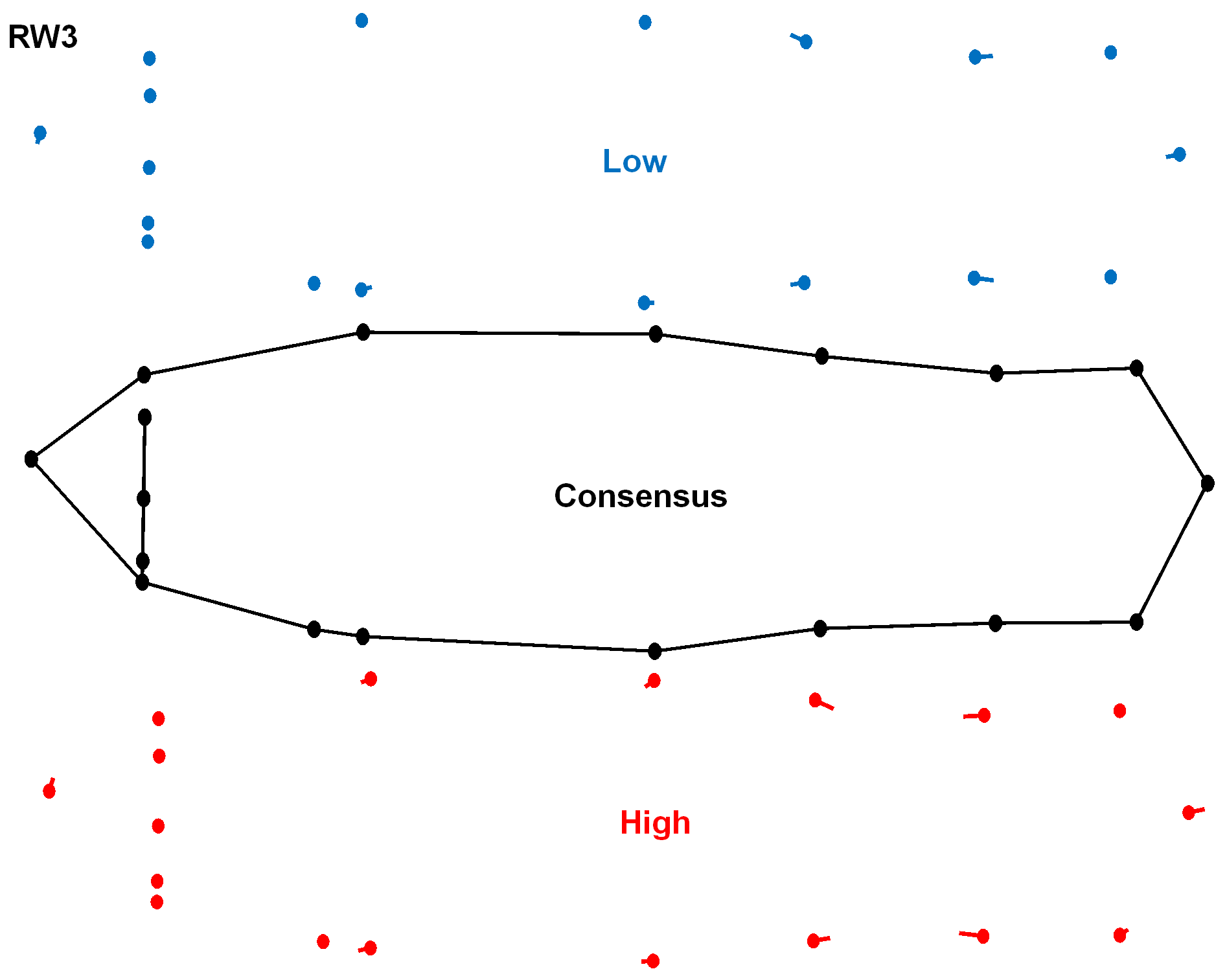
**

**Fig. S3:** The thin-plate splines illustrating shape variation between high (red dots) and low (blue dots) quantity male guppy that were captured by the relative warp (RWs) scores. RW_1_ describes variation in the shape of the abdomen (flank), RW_2_ describes variation in the shape of the gill region and operculum plate, and RW_3_ describes the elongation of males’ caudal peduncle.
